# The readiness potential reflects detailed prediction of action outcome

**DOI:** 10.1162/imag_a_00546

**Published:** 2025-04-25

**Authors:** Christoph Gärtner, Tjerk Dercksen, Andreas Widmann, Max-Philipp Stenner, Nicole Wetzel

**Affiliations:** Leibniz Institute for Neurobiology, Magdeburg, Germany; Wilhelm-Wundt-Institute for Psychology, Leipzig University, Leipzig, Germany; Center for Behavioral Brain Sciences, Magdeburg, Germany; Department of Neurology, Otto-von-Guericke University Magdeburg, Magdeburg, Germany; Center for Intervention and Research on Adaptive and Maladaptive Brain Circuits Underlying Mental Health, Jena-Magdeburg-Halle, Magdeburg, Germany; Magdeburg-Stendal University of Applied Sciences, Magdeburg, Germany

**Keywords:** readiness potential, action, predicition, ideomotor theory, omission responses, post-movement beta rebound

## Abstract

The sensory input arising from our own movements is predictable to varying degrees. This predictability plays a role in action selection, initiation, and the evaluation of action outcomes, and should therefore influence neural processing both before and after movement. Here, we examined the effect of sensory predictability on pre- and post-movement processing, as reflected in two signals in the human electroencephalogram (EEG): the*Readiness Potential*(RP) and the*post-movement beta rebound*(PMBR). Thirty-six participants performed self-paced button presses in three conditions, in which they received either highly predictable auditory feedback (always the same sound), weakly predictable auditory feedback (an unpredictable, pseudo-random sound), or no auditory feedback. In the former two conditions, 20% of button presses unexpectedly elicited no sound. In these omission trials, we observed brain responses, which indicated that participants formed sensory predictions of a varying level of detail. Crucially, RP amplitude was increased when button presses always produced the same sound, compared to pseudo-random sounds, or no sound, with the latter two conditions showing no difference. In contrast, the PMBR was unchanged across conditions. Our results indicate that the specificity of sensory prediction influences the pre-movement processes reflected in the RP, even when these details are not task-relevant.

## 1 Introduction

The sensory consequences of our own actions are often predictable, yet to varying degrees. When we use the horn of our own car, we have a specific prediction of the sound that we know the horn will produce. However, when we use the horn of a rental car for the first time, our prediction is necessarily less specific—we can expect a sound, but we cannot predict which one. In this study, we examined how sensory predictions of varying specificity interact with mechanisms underlying the preparation and evaluation of a voluntary action.

Sensory prediction plays an important role in contemporary theories of perception (de Lange et al., 2018) and motor control (Shadmehr et al., 2010). Prediction influences motor control at different stages of movement: prior to the initiation (Elsner & Hommel, 2001;Hommel, 1996;Kunde et al., 2004), during the execution (Wagner & Smith, 2008), and subsequent to the completion (Mazzoni & Krakauer, 2006) of movement. Following the ideomotor theory (Korka et al., 2022;Shin et al., 2010), anticipation of sensory action-effects before movement facilitates the selection and initiation of movement (Kunde et al., 2004), even when sensory consequences of movement are not explicitly task-relevant (Hoffmann et al., 2001). During and after movement, sensory prediction plays an important role in the evaluation of movement outcomes, and in updating of current and future movement plans (Mazzoni & Krakauer, 2006;Wagner & Smith, 2008). Similar to its influence on pre-movement processing, effects of sensory prediction on post-movement outcome evaluation exist even when sensory feedback is not task-relevant (Hoffmann et al., 2001;Ryan Morehead et al., 2017).

In the current study, we aimed to investigate the influence of sensory prediction on two physiological markers related to pre- and post-movement processing: the (pre-movement) readiness potential (RP) and the post-movement beta rebound (PMBR).

The*Readiness Potential*is a slow, negative potential shift at central electrodes in the EEG, preceding a voluntary movement by 1 to 2 seconds (Deecke & Kornhuber, 1978;Kornhuber & Deecke, 1965). The RP comprises two distinct components, an early and a late component (Deecke & Kornhuber, 1978;Kornhuber & Deecke, 1965;Shibasaki & Hallett, 2006). The early RP is thought to be generated in the supplementary motor area (SMA), the pre-supplementary motor area (pre-SMA), and cingulate motor area (Cunnington et al., 2003;Deecke & Kornhuber, 1978;Kornhuber & Deecke, 1965;Praamstra et al., 1996;Shibasaki & Hallett, 2006). The major potential shift is located at the Vertex, that is, not lateralized. In contrast, the late RP lateralizes to channels above the primary motor cortex (M1) contralateral to the moving body part (Cunnington et al., 2003;Kornhuber & Deecke, 1965;Praamstra et al., 1996;Shibasaki & Hallett, 2006).

Recent studies have shown that the RP exhibits a larger amplitude when voluntary movements produce auditory or visual feedback, compared to situations where the only sensory feedback from the movement is the inherent somatosensory feedback (Pinheiro et al., 2020;Reznik et al., 2018;Vercillo et al., 2018;Wen et al., 2018).Vercillo et al. (2018)observed a larger RP when button presses were coupled with visual feedback, compared to when they were not. Similarly,Wen et al. (2018)andReznik et al. (2018)demonstrated increased RP amplitudes when button presses generated consistent auditory feedback, compared to inconsistent feedback, or no auditory feedback. Finally,Pinheiro et al. (2020)found that even imagining auditory feedback, especially vocal sounds, enhances the RP. Together, these studies indicate that the amplitude of the RP increases when sensory feedback from a movement is predictable.

While previous studies converge on a role of the RP in coding the probability of auditory or visual feedback of imminent movement, it has remained unclear whether the RP reflects an “unspecific expectation, i.e., a general preparedness to act” (Dignath et al., 2019, p. 6), or instead specific predictions of the sensory consequences of movement. This distinction is important because the ideomotor theory predicts a role of anticipation of specific action-effects in movement generation (Stöcker et al., 2003). The ideomotor theory assumes formation of an “internalized image” of forthcoming action effects which, ultimately, acquires control over the sequence of actions (Korka et al., 2022;Shin et al., 2010). This entails “internalized images” that are sufficiently specific to differentiate between alternative actions. Whether or not the RP reflects such specificity, or merely an unspecific expectation, has remained unanswered. Here, we fill this gap by testing whether the RP amplitude is sensitive to the specificity with which action consequences can be predicted. To achieve this, we examined whether the RP amplitude is larger when an action generates a specific (and therefore a fully predictable) sound, compared to when an action generates an unpredictable sound.

A post-movement signal that has received considerable attention with respect to sensory prediction is the*Post Movement Beta Rebound*. PMBR is an increase in beta-frequency power (13–30 Hz) that starts within the first 500 ms after movement ceases, and is associated with sensory prediction and the evaluation of motor outcomes. Using EEG and fMRI, the PMBR has been localized to the sensorimotor cortex, particularly the postcentral sulcus (Jurkiewicz et al., 2006). Research byTan, Wade, et al. (2016)andPalmer et al. (2019)has demonstrated that PMBR amplitude is enhanced when participants can accurately predict movement outcomes, such as the trajectory in a visuomotor task. Additionally, studies byKorka et al. (2023)andPape and Siegel (2016)have shown that PMBR not only correlates with successful task performance, but also decision-making, highlighting its role in motor performance evaluation. In line with the theory that PMBR may reflect the precision of an internal sensorimotor model across sensory modalities (Pape & Siegel, 2016;van Kemenade et al., 2016), we hypothesized that PMBR will be stronger when an action consistently generates the same, predictable sound, compared to when it produces a variable, unpredictable sound. Furthermore, we hypothesized that PMBR will be more similar between the motor control condition and the single sound condition, compared to the random sound condition. This is because the reliability of sensory prediction in the motor control condition is high (i.e., reliable prediction of no auditory feedback), and, therefore, more similar to the single sound condition than to the random sound condition.

An elegant approach to demonstrate that humans, indeed, form sensory predictions is to unexpectedly omit predicted sensory feedback from a movement. This typically generates omitted stimulus responses in the EEG. Several studies have shown omission responses in post-movement ERPs (Dercksen et al., 2020,^2022^,^2023^;SanMiguel, Saupe, et al., 2013;SanMiguel, Widmann, et al., 2013;Stekelenburg & Vroomen, 2015), which can be separated based on their latency into components called oN1, oN2, and oP3 (Korka et al., 2022). The oN1 component is typically understood to represent the discrepancy between sensory prediction and actual sensory input (Schröger et al., 2015). Several auditory omission studies have coupled button presses either with a constant, and therefore fully predictable sound, or a relatively unpredictable sound, randomly chosen from a larger set of sounds so that sensory prediction remains unspecific, and occasionally and unexpectedly omitted sounds in both conditions (Dercksen et al., 2020,^2022^;SanMiguel, Saupe, et al., 2013;SanMiguel, Widmann, et al., 2013).SanMiguel, Saupe, et al. (2013)were able to demonstrate a stronger omission response when there was a specific prediction, whileDercksen et al. (2020)showed that a diminished, but significant omission response was also elicited in case of an unspecific prediction. This suggests the presence of both specific and unspecific predictions along the cortical hierarchy.

In this study, we sought to examine the impact of detailed sensory prediction on the RP and PMBR: First, we hypothesized that the RP’s amplitude would increase when sound identity was predictable, compared to when it was not. This would provide evidence that the RP reflects precise, specific sensory predictions rather than general anticipation of any auditory feedback. Second, building on the idea that post-movement beta power represents the precision of an internal sensorimotor model (Tan, Wade, et al., 2016), and may be part of a multi-modal predictive mechanism (Pape & Siegel, 2016;van Kemenade et al., 2016), we expected that PMBR would be lower when sound identity is unpredictable, compared to when it is predictable. Third, to verify that participants were, indeed, forming predictions regarding the upcoming stimulus in our paradigm, we also measured brain omission responses, and expected to replicate the findings of specific and unspecific predictions encoded in omission responses (Dercksen et al., 2020,^2022^).

## 2 Methods

### 2.1 Participants

We collected EEG data from 40 participants aged between 20 and 28 (mean age = 24.6, SD = 1.7, 50% female, 50% male). Participants confirmed that they had no history of neurological diseases and had non-impaired, or corrected-to-normal, vision, as well as non-impaired hearing. All were right-handed, as shown by an adapted version of the Edinburgh Handedness Inventory (Oldfield, 1971). Participants gave their written informed consent to participate in the study. They received either reimbursement (10 EUR per hour) or university credit points. We included 36 participants (mean age = 24.5, SD = 1.9, 50% female, 50% male) in the analysis as we faced technical problems (Bluetooth connection loss during recording) with two participants, and two participants fell asleep during the experiment. The project was approved by the local ethics committee.

### 2.2 Stimuli, apparatus, and data acquisition

As sound stimuli, we used 48 different common environmental sounds, such as a car horn or a barking dog. The sounds were the same as in earlier studies (Dercksen et al., 2020,^2022^,^2023^,^2024^;SanMiguel, Saupe, et al., 2013;SanMiguel, Widmann, et al., 2013). The sounds were judged as identifiable in an earlier study (Wetzel et al., 2011), and were processed with a 10 ms rise and fall time tapered-cosine window. RMS matching was applied to ensure consistent loudness. The loudness level for all participants was set at 80.5 dB SPL. Participants heard the sounds binaurally via headphones (Sennheiser HD – 25 Headphones; Sennheiser, Germany).

Participants were seated in an upright position in front of a laptop (AsusTek GL752VW, display size: 43,9 cm (17,3”), resolution: 1920 x 1080 (16:9), frame rate: 60 hz). The laptop screen showed a white fixation cross (0.67° x 0.67° visual angle) on a gray background. The distance between the screen and the participant was approximately 60 cm. Participants placed their right index finger on a custom-built infrared photoelectric button padded with sound-absorbing material, which elicited no audible sound itself when pressed.

Participants were fitted with a scalp EEG recording with a total of 33 active electrodes (incl. reference and ground electrodes), arranged according to the international 10-20 system. Additionally, EOG was recorded by two electrodes next to the right and left outer canthi of the eyes and below the left eye. A reference electrode was placed on the tip of the nose, and a ground electrode at Fpz. The recording setup employed a LiveAmp amplifier from BrainProducts (Gilching, Germany). The recording was conducted using BrainVision Recorder software (version 1.21) at a sampling rate of 250 Hz, with DC coupling and a 65 Hz low-pass filter. The experiment was programmed using*Psychtoolbox*(version 3.0.15,Kleiner, Brainard, & Pelli, 2007) and was run on GNU Octave (Ubuntu 22.04.1 LTS).

### 2.3 Tasks and procedures

**Fig. 1.**
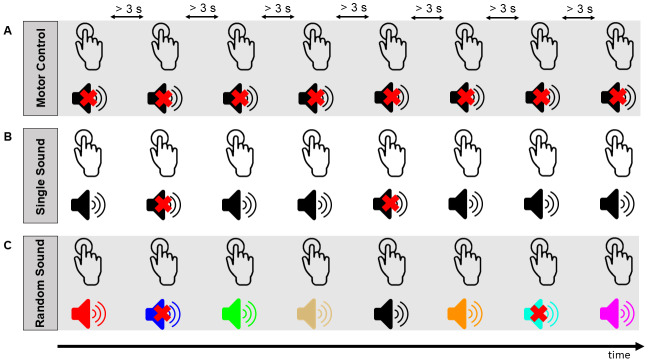
Schematic of the paradigm. Panels (A) to (C) illustrate three experimental conditions in which participants performed self-paced button presses with their right index finger, maintaining a minimum inter-press interval (IPI) of 3 seconds. A white fixation cross provided visual feedback, turning red if the IPI was below 2700 ms. Panel (A) illustrates the motor control condition, in which button presses produced no sound. Panel (B) and Panel (C) illustrate the single sound condition and random sound condition, respectively, in which the button press frequently generated a sound (in 80%). In 20% of trials, no sound was presented to induce a prediction error (indicated by crossed out speakers). The color of the speaker represents a unique sound (including black). In the single sound condition (panel B), every button press that was not part of an omission trial generated the same sound, which was, therefore, highly predictable. In the random sound condition (panel C), every button press that was not part of an omission trial generated a sound that was pseudo-randomly selected from a set of 48 sounds. Participants could, therefore, form no accurate prediction about the identity of the upcoming sound.

Figure 1shows a schematic of the set up and paradigm. The task was to press a button with the right index finger at a self-paced rate, however, with a minimum*inter-press interval*(IPI) of 3 seconds. We chose this interval because it was large enough to allow for a slow build-up of the RP before the button press. Participants received instructions to keep their finger on the button between presses. They were asked to maintain their focus of attention on a white fixation cross displayed in the middle of the screen. To provide trial-wise feedback about the IPI, the fixation cross changed from white to red for 1 second whenever the IPI fell below 2700 ms.

We defined three conditions, similar toDercksen et al. (2020),SanMiguel, Saupe, et al. (2013), andSanMiguel, Widmann, et al. (2013): a single sound condition (SS), a random sound condition (RS), and a motor control condition (MC). In the two sound conditions (SS and RS), 80% of button presses were followed by a sound. There was a delay of 4.5 ms between button press and sound onset caused by the button box and sound card latency. In 20% of the trials, no sound was presented in response to a button press (omission trials). This was included to evoke a prediction error reflected by omission responses.

In the single sound condition, every button press that was not part of an omission trial generated the same sound. Each participant completed several blocks of the single sound condition. In different blocks, different sounds were presented. In the random sound condition, every button press that was not part of an omission trial generated a sound that was chosen from a set of 48 sounds. Within each random sound block, the 48 sounds were shuffled pseudo-randomly, such that each sound was played once before any was repeated. Each time all 48 sounds had been repeated, they were reshuffled and played again in a new random order. This process minimized the likelihood of participants being able to accurately predict the sequence of sounds. Each participant completed several blocks of the RS condition. In the single sound condition and the random sound condition, 20% of button presses did not generate a sound. The position of these omission trials within a block was pseudo-randomly determined, with two constraints: the first five trials of each block could not be omission trials, and the two trials following an omission trial could not be omission trials. In the motor control condition, button presses never generated any sound.

The experiment was divided into three identical parts, with a short break in between consecutive parts. The duration of breaks was self-paced with a limit of five minutes, which was rarely exceeded (3’28” on average). In each part, participants completed one motor block, two single sound blocks, and two random sound blocks. The block order was pseudo-randomized based on subject index, with odd-indexed subjects receiving one sequence (part 1: MC, SS, RS, SS, RS; part 2: SS, RS, MC, SS, RS; part 3: SS, RS, SS, RS, MC) and even-indexed subjects another (part 1: MC, RS, SS, RS, SS; part 2: RS, SS, MC, RS, SS; part 3: RS, SS, RS, SS, MC). One motor control block consisted of 50 trials, for a total of 150 control trials across the entire experiment. Each single and random sound block consisted of 75 trials, including 15 trials with omission trials, for a total of 360 sound trials and 90 omission trials per sound condition across the entire experiment.

Ahead of the actual recording, participants practiced pressing the button with the required minimum IPI. To do so, they received visual feedback on the screen showing the time in between button presses. Each participant completed two training blocks with 30 trials each.

At the start of each block, participants placed their right index finger on the button, and looked at the fixation cross on the screen for 10 seconds. This was done with the intention to set a common baseline for the analysis.

### 2.4 EEG analysis

Data were analyzed using*EEGLAB*(Delorme & Makeig, 2004) and*Fieldtrip*(Oostenveld et al., 2011) in MATLAB (2021a, 2021b, 2022a). To accommodate the different time windows, spectral composition, and spatial distributions we expected for the RP, omission responses, and PMBR, respectively, our pre-processing pipeline was tailored to each signal of interest.

#### 2.4.1 Readiness potential

For the analysis of the RP, we filtered the continuous data offline using a 48 Hz low-pass filter (Kaiser windowed sinc FIR filter, order 402, beta 5, transition bandwidth 4 Hz). No high-pass filter was applied to avoid distorting the RP, given that its spectral composition includes low frequencies. From the filtered, continuous data, we removed noisy channels, defined as channels for which the robust z-score of the standard deviation of voltages over time exceeded a threshold of 3 (Bigdely-Shamlo et al., 2015). Trials for further pre-processing were defined from -2700 to 500 ms around the button press, with the remaining data outside these intervals being trimmed to maintain a continuous dataset. In a first, coarse trial rejection, we removed trials in which the signal changed by at least 600 μV (difference between maximum and minimum voltage). In addition, we removed trials with an IPI less than 2700 ms, or more than 6000 ms, as well as the first five trials of each block and the two trials following omission events. The latter two criteria were included to exclude potential effects of shifts in attention.

Artifacts caused by ocular movement and heartbeat were removed via ICA. To improve ICA decomposition, independent components were computed from a copy of the continuous data that was high-pass filtered at 1 Hz (Kaiser windowed sinc FIR filter, order 1604, beta 5, transition bandwidth 1 Hz), and low-pass filtered at 48 Hz (Kaiser windowed sinc FIR filter, order 402, beta 5, transition bandwidth 4 Hz) (Winkler et al., 2015). The resulting unmixing matrices were then applied to the less stringently filtered dataset, which was further analyzed, while the high-pass filtered dataset was discarded. In this way, the ICA received input data optimized for the detection of ocular artifacts (i.e., after high-pass filtering) (Winkler et al., 2015), while the data that we eventually analyzed were not high-pass filtered, avoiding potential signal distortions due to filtering (Acunzo et al., 2012;Widmann et al., 2015). Aiming to remove all artefacts caused by eye movements, blinks, and heart artefacts, we used the*IClabel*plugin (Pion-Tonachini et al., 2019), which is an automatic classification algorithm that provides an indication of what each component represents. In addition, components were classified independently by two raters (CG and TD), followed by a consensus about which components to remove. On average, 10 components were removed (SD = 1.6, range: 7-13 components).

After ICA component removal, previously removed channels were interpolated, using spherical interpolation with the default settings provided by EEGLab. Epochs of interest were then defined, which covered a period from -1900 ms to 500 ms relative to the button press. In a second trial rejection step, we removed epochs exceeding a 225 μV signal-change threshold. On average, 216 trials (SD = 15, range = 180–238) were retained in the single sound condition, 218 trials (SD = 18, range = 165–238) in the random sound condition, and 120 trials (SD = 12, range = 86–134) in the motor control condition.

Data were baseline corrected by subtracting the mean amplitude between -1900 ms to -1700 ms. To establish the baseline period, we adopted Steven Luck’s approach (Luck, 2005), which identifies the time point at which the grand-average time-course clearly deviates from a flat line for the first time. We selected as a baseline the last 200 ms before that time point (Luck, 2005). Pre-processed, epoched data were then averaged for each subject and condition separately, time-locked to the button press.

#### 2.4.2 PMBR

For the analysis of the PMBR, we filtered the data offline with a bandpass filter from 1 Hz to 100 Hz (Kaiser windowed sinc FIR filter, order 802). We used the same approach for removing noisy channels, for a first, coarse trial rejection, for the ICA, and for interpolating noisy channels as described for the RP, with the exception that no copy of the continuous data was needed for the ICA, given the initial bandpass filter used to analyze the PMBR. Epochs were defined from -2700 ms to 2700 ms relative to the button press. On average, 10 independent components were removed (SD = 1.6, range 5–15). The ICA was followed by a second trial rejection step, in which epochs exceeding a 200 μV signal-change threshold were removed. On average, 118 trials (SD = 25, range: 58-147) were retained in the single sound condition, 118 trials (SD = 26, range: 49-150) in the random sound condition, and 100 trials (SD = 27, range: 30-134) in the motor control condition. For omission trials, there were an average of 70 trials (SD = 15, range: 26–89) in the single sound condition and 70 trials (SD = 15, range: 31–90) in the random sound condition.

We then performed a time-frequency decomposition in Fieldtrip (mtmconvol), for which we used a*Hanning taper*of 400 ms. Power was analyzed between 2.5 and 47.5 Hz, in steps of 2.5 Hz, evaluated every 40 ms. A baseline correction was performed by computing relative change in power with reference to the mean power across an entire epoch, averaged for each condition separately (Tan et al., 2014;Torrecillos et al., 2015). We opted against utilizing the initial 10-second waiting periods as a baseline window at the start and end of each block, as we discovered substantial differences in the power spectrum between the resting state and the subsequent task engagement across the entire frequency range.

To identify channels that displayed the lateralized beta-power modulations that are typical for movement-related activity, we segmented the timeline from -1600 ms to +2000 ms around the button press into consecutive 200 ms intervals. We then calculated the mean change in beta power (12.5 to 32.5 Hz) from baseline, across all conditions, for each of these 200-ms time windows. The resulting series of topographies of beta-power changes across time are presented inFigure 2A. This procedure revealed that channels CP1, Cz, C3, and FC1 showed the expected pre- and post-movement, lateralized modulation of beta-power. These channels, therefore, served as channels of interest.

**Fig. 2.**
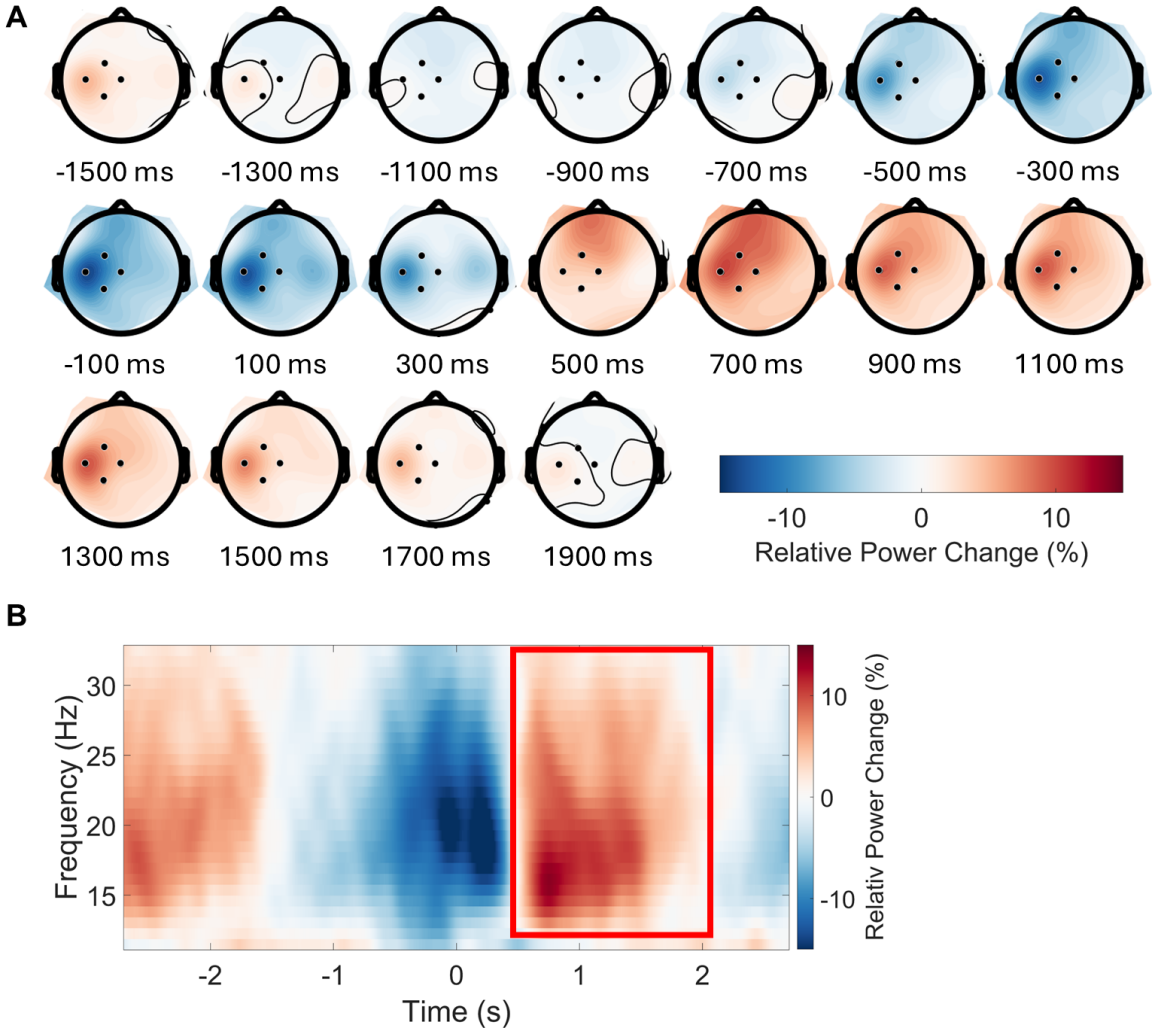
Panel (A) displays topographic plots illustrating the relative change in power (in percentage) averaged across the beta frequency band (12.5 Hz to 32.5 Hz) and all conditions, in consecutive time bins (200 ms bin width) between -1600 ms to 2000 ms. Bins were centered on the time points indicated below each topographic plot. Baseline correction was performed by calculating the relative change in power, using the mean power across the entire epoch as a baseline. This analysis identified channels C3, CP1, Cz, and FC1 (highlighted) as having the most pronounced increase in post-movement beta power. Panel (B) presents a time-frequency plot with data averaged across all conditions and these channels of interest (C3, CP1, Cz, and FC1). The red rectangle represents the time-frequency window (500 ms to 2000 ms, and 12.5 to 32.5 Hz) chosen for statistical analysis.

In these channels of interest, post-movement beta power increased above baseline from 500 ms to 2000 ms after the button press (Fig. 2B). We, therefore, calculated PMBR as the mean change in power from baseline across the four channels of interest, our a priori defined frequency window of interest (12.5 to 32.5 Hz), and this time window of interest (500 to 2000 ms), for each subject and condition separately.

#### 2.4.3 Omitted stimulus responses

For the analysis of the post-stimulus ERP, we followed the pre-processing pipeline as it was performed in the study byDercksen et al. (2020). We filtered the raw data offline with a 0.1 Hz high-pass filter (Kaiser windowed sinc FIR filter, order 8024, beta 5, transition bandwidth 0.2 Hz) and a 48 Hz low-pass filter (Kaiser windowed sinc FIR filter, order 402, beta 5, transition bandwidth 4 Hz). We used the same approach for removing noisy channels, for a first, coarse trial rejection, for conducting the ICA, and for interpolating noisy channels as described for the RP. Epochs were defined from -2700 to 500 ms. After completing the ICA based on the 1 Hz-filtered copy of the data, the selected components were removed from the 0.1 Hz high-pass filtered dataset. On average, 10 components were removed (SD = 1.6, range: 7-13 components). Epochs of interest were defined as starting 200 ms before the button press and ending 500 ms after the button press. We removed epochs exceeding a 125 μV signal-change threshold. Data were baseline corrected by subtracting the mean amplitude between -200 ms to -100 ms. On average, 82 omission trials (SD = 4.9, range = 71-89) were available for analysis in the single sound condition, 83 omission trials (SD = 5.9, range = 67–90) in the random sound condition, and 122 trials in the motor condition (SD = 10.7, range 87 = 87–135).

In line with previous auditory omission research (Dercksen et al., 2020,^2022^,^2024^;Korka et al., 2020), we conducted a temporal PCA. PCA provides a solution to the limitations of traditional ERP analysis by deconstructing the ERP waveform into its constituent elements through a factor analysis method, as the observed peaks in the traditional ERP analysis are not reliable indicators of its underlying components (Scharf et al., 2022). This technique provides a more accurate reflection of the underlying components than the observable peaks of the ERP waveform. Additionally, using PCA facilitates a consistent and objective comparison of omission responses across different studies, supporting its use in both current and previous auditory omission research such as the work byDercksen et al. (2020,^2022^,^2024^) andKorka et al. (2020).

We conducted temporal PCAs on the individual-subject, trial-averaged ERP data, which included one average waveform per participant, condition, and electrode. To determine the number of components to retain, we employed Empirical Kaiser Criterion (EKC), which retains only those components with eigenvalues greater than 1. The eigenvalue is a measure of the amount of variance explained by each principal component. If the eigenvalue is greater than 1, it implies that the principal component accounts for more variance than a single original variable, and it is considered worth retaining (Braeken & Van Assen, 2017;Scharf et al., 2022). To implement the PCA, we used an R (version 4.1.2) implementation of the Geomin rotation method with ε = 0.01, following the procedure outlined in the tutorial byScharf et al. (2022). The Geomin rotation method is preferred in this context as it is less likely to merge components (representing separate phenomena) that exhibit strong temporal and spatial overlap compared to other rotation methods, like Promax (Scharf & Nestler, 2018,^2019^). One PCA was computed for omission trials in each condition (single sound condition, random sound condition), and for the motor control condition, with the aim to extract the oN1, oN2, and oP3 components. Based on the distinct topographies observed for each component, specific regions of interest (ROIs) or single channels were identified for subsequent statistical analysis.

### 2.5 Statistics

#### 2.5.1 RP

We performed a non-parametric cluster-based permutation test as implemented in*Fieldtrip*(Maris & Oostenveld, 2007). Given that the RP is the most pronounced in central channels (Shibasaki & Hallett, 2006), channels of interest for the cluster-based permutation test included*FC1, FC2, C3, C4, Cz, CP1*, and*CP2*. We tested a time window from -1700 ms to 0 ms, covering the period from the end of the baseline phase up to the moment of the button press. In a first step, the EEG amplitudes of all subjects at each channel and time point were compared between two conditions (Single Sound vs. Random Sound, Single Sound vs. Motor Control, Random Sound vs. Motor Control; pairwise dependent-samples*t*-tests between conditions). Channel × time data points exceeding a threshold of*α*< 0.05 for the*t*-statistic were selected. Clusters were formed across neighboring time points and channels that exceeded this threshold (separately for positive and negative*t*-values), and a cluster-level statistic was obtained by summing all*t*-values in each cluster. A*p*-value was computed for each cluster using the*Monte Carlo method*, for which the described procedure to obtain the cluster-level statistic was repeated for 1000 permutations of the condition labels. This provided a non-parametric distribution of the cluster-level statistic under the null hypothesis of no condition difference (i.e., exchangeability of condition labels). A*p*-value was obtained for each cluster observed in the original data as the proportion of permutations for which the largest observed cluster-level statistic exceeded the cluster-level statistic observed in the original data.*p*-values < 0.025 were taken as evidence that condition differences were significant (two-sided test).

In instances where no significant clusters comparing two conditions could be identified by the cluster-based permutation test, an additional Bayesian paired-samples t-test was conducted to further assess the strength of evidence for differences in specific measures between those conditions. For this, we computed average RP amplitudes, across the channels and time points for which we observed significant differences between the other conditions. (i.e., across channels and time points that formed the clusters associated with a significant*p*-value in the cluster-based permutation test).

We adopted the interpretation framework for Bayes factors as outlined byLee and Wagenmakers (2013). They categorize Bayes factors into specific ranges: “anecdotal” for 0.33–3, “moderate” for 3–10 or 0.33–0.1, “strong” for 10–30 or 0.1–0.033, and “very strong” for values greater than 30 or less than 0.033. We substituted the word “anecdotal” with “weak”, and “very strong” with “decisive”, to make the interpretation clearer.

#### 2.5.2 PMBR

We compared PMBR between conditions in two one-way repeated-measures ANOVAs. A first ANOVA tested for differences between the two sound conditions, and between each sound condition and the motor control condition, while the second tested for differences between the omission trials of each sound condition, and between omission trials and the motor control condition.

We also performed a cluster-based permutation test as implemented in Fieldtrip (Maris & Oostenveld, 2007) to test for differences between conditions. The time- and frequency-windows of interest for the cluster-based permutation test ranged from 500 ms to 2000 ms, and from 12.5 to 32.5 Hz, respectively. We also preselected the channels of interest identified via the approach outlined inFigure 2(CP1, Cz, C3, and FC1).

#### 2.5.3 Omitted stimulus responses

We compared each omitted stimulus response component (oN1, oN2, and oP3) across conditions in a one-way repeated-measures ANOVA with three levels (single sound omission, random sound omission, motor control). Following this, we computed post hoc pairwise*t*-tests with Holm’s correction for the oN1, oN2, and oP3. We conducted Bayesian paired-samples*t*-tests to determine the strength of evidence against a difference between conditions.

## 3 Results

### 3.1 Behavioral results

Participants maintained the required pace across the three conditions. Average IPIs were 3292 ms (SD 366 ms) in the single sound condition, 3290 ms (SD 321 ms) in the random sound condition, and 3409 ms (SD 457 ms) in the motor control condition (median across trials, followed by mean across subjects). An ANOVA revealed no significant difference in median IPI between conditions (F = 1.145,*p*= 0.332, η² = 0.021). Bayesian pairwise t-tests provided anecdotal evidence against any difference in IPI between the motor control and sound conditions, and moderate evidence against any difference between the two sound conditions (MC vs. SS:*BF_10_*= 0.455, MC vs. RS:*BF_10_*= 0.495, SS vs. RS:*BF_10_*= 0.243).

### 3.2 Readiness potential

**Fig. 3.**
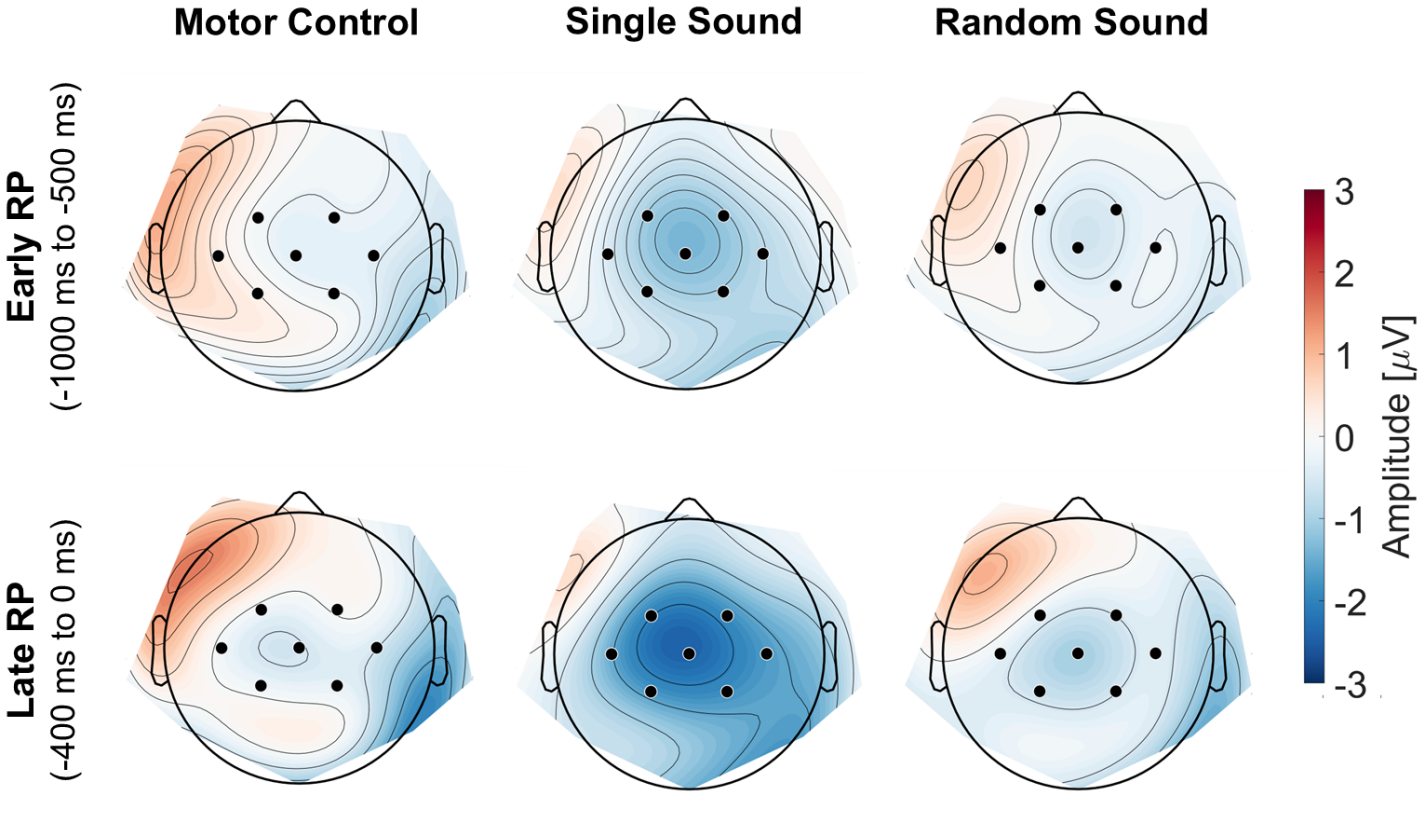
Topographical maps of the RP across conditions. The upper row shows the early RP averaged from -1000 ms to -500 ms, while the lower row presents the late RP averaged from -400 ms to the moment of the button press. Channels of interest (C3, C4, Cz, CP1, CP2, FC1, and FC2) are highlighted.

Figure 3displays the topography of the RP, averaged between -1000 ms and 0 ms, for each condition.

**Fig. 4.**
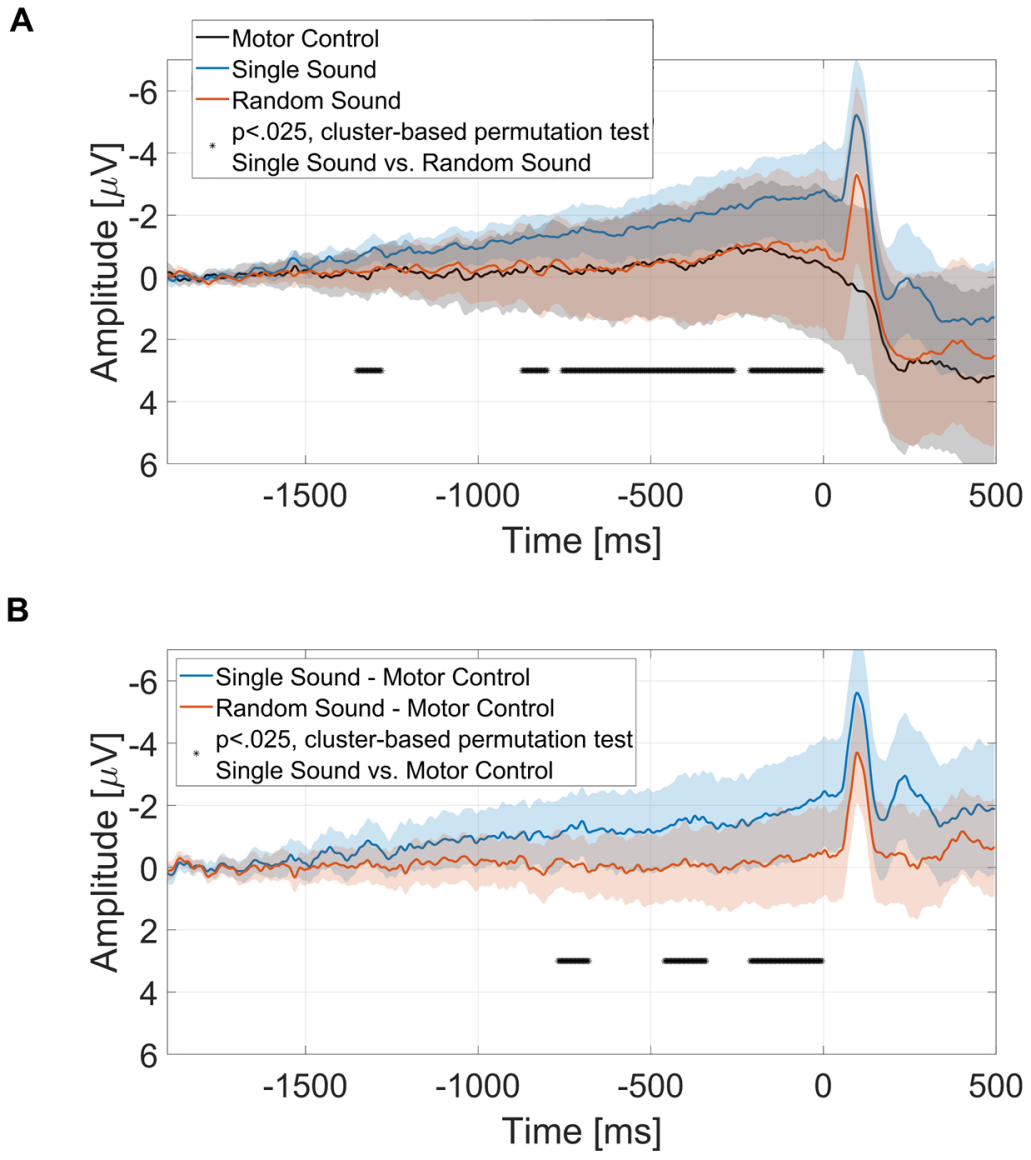
Grand-average readiness potential and difference waves at the channels of interest (*C3, C4, Cz, FC1, FC2, CP1, CP2*), baseline -1900 to -1700 ms. After selecting a time window of interest (-1700 ms to 0 ms), cluster-based permutation testing revealed a difference between SS and RS, as well as SS and MC. Asterisks (*) mark the time points included in clusters associated with a significant*p*-value. Shaded colors show 95% CI of corresponding conditions. Panel (A) shows grand-average readiness potential in all three conditions. Asterisks represent time points associated with a significant*p*-value comparing SS and RS. Panel (B) shows grand-average difference waves between each sound condition and the motor control condition. Asterisks represent time points associated with a significant*p*-value comparing SS and MC.

Cluster-based permutation testing with an a priori defined time window (-1700 to 0 ms), and a priori defined channels of interest (*C3, C4, Cz, CP1, CP2, FC1*, and*FC2*), revealed significant differences between the single sound and random sound condition (four clusters). Clusters included all seven channels of interest, and time intervals ranging from -1352 to -1280 ms, -872 to -796 ms, -756 to -264 ms, and from -212 to 0 ms.Figure 4displays the grand-average RP for the single sound, random sound, and motor control condition, averaged across channels of interest (*C3, C4, Cz, CP1, CP2, FC1*, and*FC2*). Black asterisks mark the time points belonging to clusters associated with a significant*p*-value.

Cluster-based permutation testing also revealed a significant difference between the single sound condition and motor control condition (three clusters). Clusters included time intervals ranging from -768 to -680 ms, -460 to -340 ms, and from -212 to 0 ms.

Comparing the motor control condition to the random sound condition, we found no significant differences. Bayesian paired-samples*t*-tests were conducted to compare RP amplitude between the motor control and random sound condition, focusing on the channels of interest and the three time intervals in which the single sound condition differed significantly from the motor control condition. Bayes factors indicated moderate evidence against a difference between the random sound condition and the motor control condition (cluster 1: BF_10_= 0.204, cluster 2: BF_10_= 0.196, and cluster 3: BF_10_= 0.194).

### 3.3 Post movement beta rebound

**Fig. 5.**
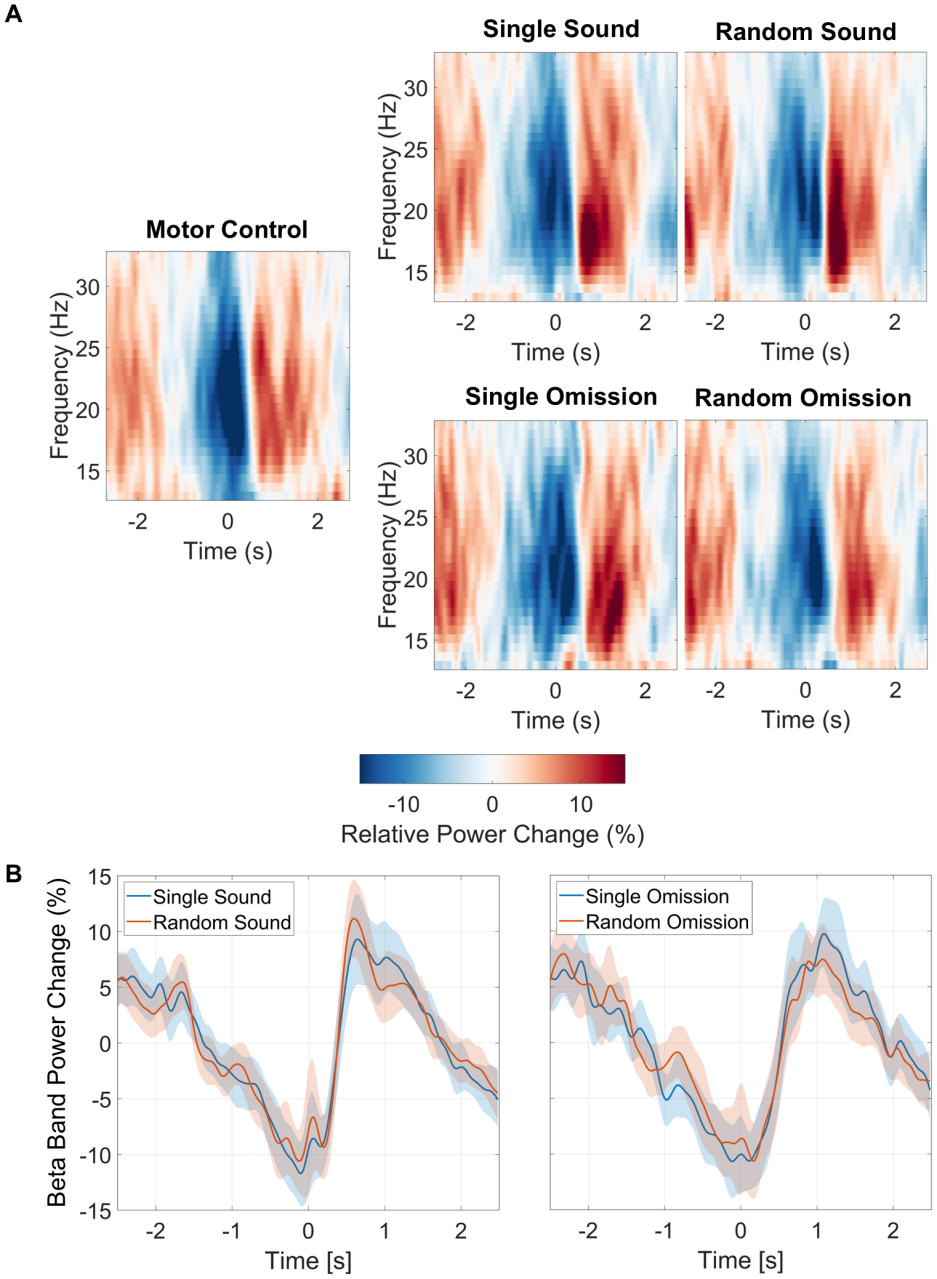
(A) Relative power change by conditions at C3, Cz, FC1, and CP1 in the beta band frequency range [12.5 Hz–32.5 Hz]. (B) Beta-band change in power, in percent. Shaded colors show 95% CI. Baseline correction was performed by computing relative change in power with reference to the mean power across an entire epoch, averaged for each condition separately.

Figure 5displays the percentage change in power for the single sound condition and random sound condition at channels of interest (CP1, Cz, C3, and FC1). The results of the “sound” repeated-measures ANOVA indicated no significant main effect of condition (F(2,70) = 0.221,*p*= 0.802,*η²*= 0.006). The “omission” repeated-measures ANOVA, testing for differences between the omission trials in each sound condition and the motor control, revealed a trend-level effect of condition (F(2,70) = 3.091,*p*= 0.052, η² = 0.081). Following up on this trend with post-hoc*t*-tests with Holm’s correction, we found a marginally significant difference between the single omission condition and the random omission condition (*t*= 2.456,*p*= 0.049). However, a cluster-based permutation test across the time-frequency window of interest (500 ms to 2000 ms, and 12.5 to 32.5 Hz) revealed no significant differences between conditions.

### 3.4 Omitted stimulus response

Our goal in analyzing omitted stimulus responses was to confirm that participants were, indeed, forming predictions in our paradigm, and to replicateDercksen et al.’s (2020,^2022^) findings of specific and unspecific predictions. A total of 13 components were identified through PCA of the omitted stimulus responses. Four PCA components and their corresponding topography are displayed inFigure 6. The presented components were selected by visual inspection and judged by their likelihood to reflect previously described omission responses, based on their topographies and latencies. The statistical results are summarized inTable 1. We identified the following four components of interest.

**Fig. 6.**
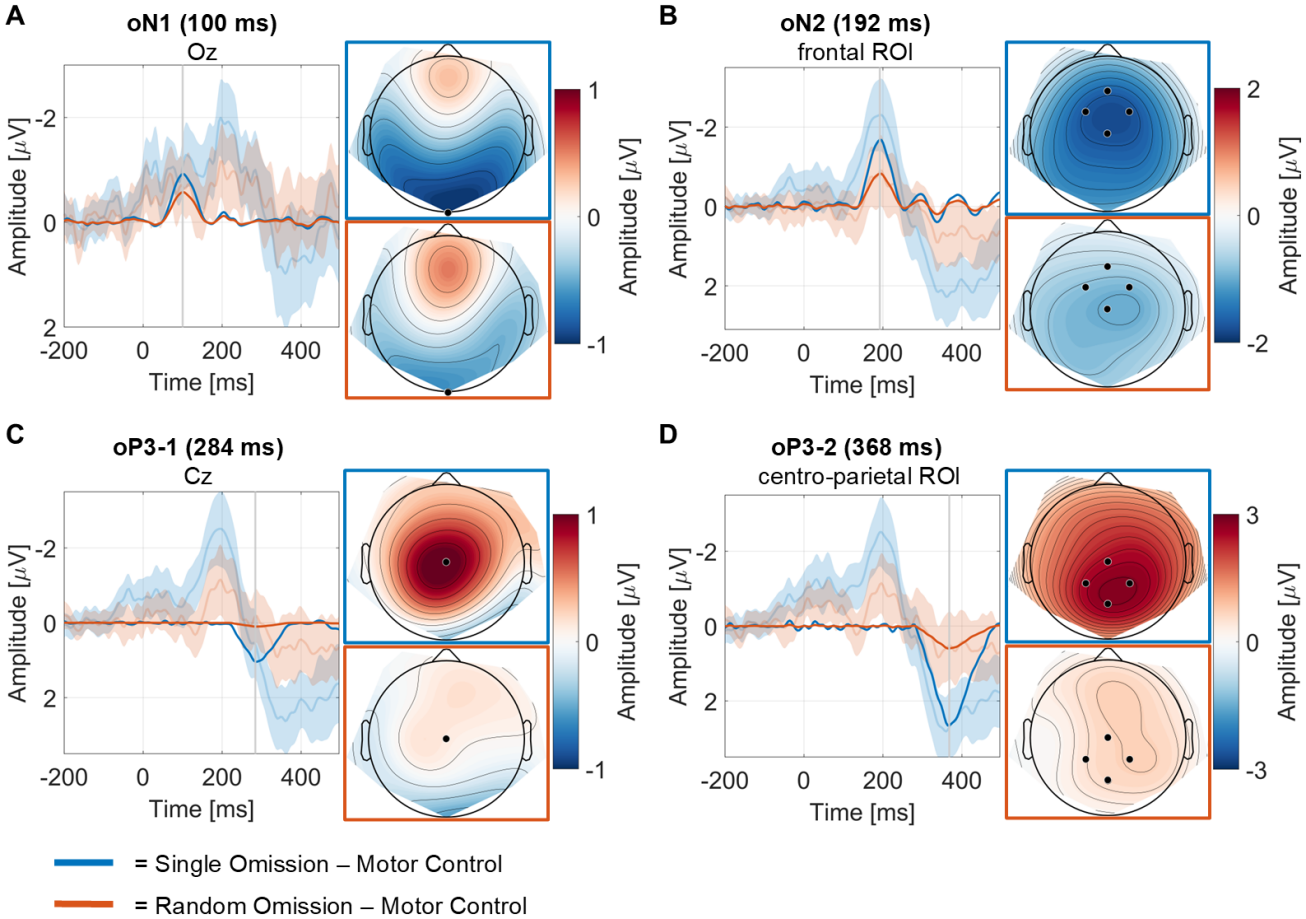
Omission responses identified using temporal PCA presented in chronological order (A-D). PCA component waveforms are represented in μV units and reflect the relative contribution of each component to the observed ERP responses. The difference waveforms between omission conditions and motor control conditions, separately for omission trials in the single sound condition and the random sound condition, are shown in opaque colors without shading. Grand-average ERPs, including 95% confidence intervals, shown in semi-transparent colors for comparison. Difference component topographies at peak latencies are shown for the single sound condition (blue frame) and the random sound condition (red frame).

**Table 1.** Summary of the statistical results based on the PCA analysis.

Omitted stimulus responses						
Repeated measures ANOVA						
	F(2,70)		*p*		η²	
oN1	7.926		<0.001		0.185	
oN2	12.826		<0.001		0.268	
oP3-1	4.872		0.010		0.122	
oP3-2	20.252		<0.001		0.367	
oP3-3	7.536		0.001		0.177	

Post-hoc tests						
	Contrast	t (35)	d		*p*_holm_	
oN1	SO vs. RO	-1.483	-0.298	0.143	BF _10_	0.432
	SO vs. MC	-3.941	-0.793		<0.001	
	RO vs. MC	-2.458	-0.495		0.033	
oN2	SO vs. RO	-2.184	-0.440		0.032	
	SO vs. MC	-5.050	-1.017		<0.001	
	RO vs. MC	-2.865	-0.577		0.011	
oP3-1	SO vs. RO	2.554	0.391		0.026	
	SO vs. MC	2.831	0.433		0.018	
	RO vs. MC	0.277	0.042	0.782	BF _10_	0.189
oP3-2	SO vs. RO	4.539	0.711		<0.001	
	SO vs. MC	6.133	0.960		<0.001	
	RO vs. MC	1.594	0.250	0.116	BF _10_	0.570

SO = single condition omission; RO = random condition omission; M = motor control.

**oN1.**Component 8, explaining 4.3% of variance, peaked at 100 ms, thus likely reflecting oN1 (Dercksen et al., 2020;^2022^). However, its topography, with a peak at electrode Oz, differed from the typical topography of the oN1 described in the literature (Dercksen et al., 2020,^2022^;Korka et al., 2020;SanMiguel, Saupe, et al., 2013) (seeSection 4). A repeated-measures ANOVA revealed a significant main effect of condition (*F*(2,70) = 7.926,*p*= < 0.001,*η²*= 0.185). This main effect was due to a difference between the motor control condition on the one hand, and the single sound condition and random sound condition on the other hand. When computing difference waves between each of the two sound conditions and the motor control condition, both the single sound condition and the random sound condition showed oN1 responses to sound omissions. A Pairwise Bayesian*t*-test indicated weak evidence (BF_10_= 0.432) against a difference between the single and random sound conditions.

**oN2.**Component 6, explaining 5.5% of variance, peaked at 192 ms over fronto-central ROI (Cz, Fz, FC1, FC2), likely reflecting oN2 (Dercksen et al., 2020,^2022^). A repeated-measures ANOVA revealed a significant main effect of condition (*F*(2,70) = 12.826,*p*= < 0.001,*η² *= 0.268), due to a difference between the motor control condition on the one hand, and the single sound condition and random sound condition on the other hand. When computing difference waves between each of the two sound conditions and the motor control condition, we observed an oN2 component both in the single sound condition and in the random sound condition. We found evidence for a more negative amplitude in the single sound condition compared to the random sound condition.

**oP3-1, oP3-2.**PCA revealed two potential components in the oP3 time range, likely reflecting distinct parts of the oP3 (Dercksen et al., 2020,^2022^). Component 3, explaining 14.5% of variance, peaked at 284 ms over electrode Cz*,*and likely reflected oP3-1. A repeated-measures ANOVA revealed a significant main effect (*F*(2,70) = 4.872,*p*= 0.010,*η²*= 0.122) of condition. Statistical analysis showed evidence for an oP3-1 in the single sound condition, but not in the random sound condition. A Pairwise Bayesian*t*-test indicated moderate evidence (BF_10_= 0.189) against an oP3-1 in the random sound condition. Component 1, explaining 23.4% of variance, peaked at 368 ms over centro-parietal electrodes (Cz, CP1, CP2, Pz), and presumably reflected oP3-2. A repeated-measures ANOVA revealed a significant main effect of condition (*F*(2,70) = 20.252,*p*= <.001,*η²*= 0.367). Statistical analysis showed evidence for an oP3-2 in the single sound condition. A Pairwise Bayesian*t*-test indicated weak evidence (BF_10_= 0.570) against an oP3-1 in the random sound condition.

In sum, significant main effects were observed in three key components—oN1, oN2, and oP3. Both the oN1 and oN2 showed larger responses in both sound conditions compared to the motor control condition, while the oP3 subcomponents only indicated a larger difference in the single sound condition. In our study, a stronger omission response was found for oN2 in the single sound condition compared to the random sound condition, suggesting the presence of specific predictions.

## 4 Discussion

Our results reveal that the RP is larger in amplitude when subjects can predict that their movement generates a specific sound, compared to a sound whose identity cannot be predicted. This specificity in predicting action consequences is also evident in the post-movement time period, specifically in the amplitude of omission responses. In contrast, PMBR shows no modulation by the degree of specificity with which auditory consequences of movement can be predicted.

### 4.1 Omission responses confirm presence of specific and unspecific predictions

Our experimental paradigm followed the design of previous omission response studies bySanMiguel, Saupe, et al. (2013),SanMiguel, Widmann, et al. (2013), andDercksen et al. (2020,^2022^). An unexpected omission in an established sequence of tones, each generated by a movement, elicits an omission response in the post-stimulus ERP. This omission response typically consists of an oN1, oN2, and oP3, each presenting with distinct and fairly consistent topographies (Dercksen et al., 2020,^2022^,^2023^;SanMiguel, Saupe, et al., 2013;SanMiguel, Widmann, et al., 2013;Schröger et al., 2015;Stekelenburg & Vroomen, 2015). The oN1 component is typically regarded as a sensory prediction error signal that occurs when expected stimuli are omitted (Dercksen et al., 2020,^2022^;SanMiguel, Saupe, et al., 2013;SanMiguel, Widmann, et al., 2013;van Laarhoven et al., 2017). The following components, oN2 and oP3, have been linked to higher-level prediction error processing, error monitoring, and higher-order cognitive processing (Dercksen et al., 2020,^2024^;SanMiguel, Saupe, et al., 2013;van Laarhoven et al., 2017). Since our primary objective concerning omission responses was to confirm specific and unspecific predictions within our paradigm, we have chosen not to delve into a detailed interpretation of each component. Instead, we refer readers to recent omission studies where these aspects are thoroughly discussed (seeDercksen et al., 2020,^2022^,^2023^,^2024^;Korka et al., 2020,^2022^;SanMiguel, Saupe, et al., 2013;SanMiguel, Widmann, et al., 2013).

However, some notable differences were observed in the current study compared to previous omission studies. Specifically, while the oN1 is typically strongest over temporal electrodes, we observed a more posterior topography. Moreover, contrary toDercksen et al. (2020), an oN2, but no oP3-1 and oP3-2, was observed in the random sound condition. Several changes to the experimental set-up could have caused the observed discrepancy with earlier studies. First, the length of the IPI was at least 3 seconds in the present study, compared to 600 to 1200 ms in the original study. Second, the proportion of omissions was 20%, not 12%, as inDercksen et al. (2020). Third, the longer IPI resulted in less omission trials being presented compared to earlier studies, possibly resulting in a decreased signal-to-noise ratio per participant. Indeed, Bayes factors indicated the need for more data for some components in order to draw more convincing conclusions. This provides important information for future omission research, which could directly examine the influence of IPI and omission likelihood on EEG omission responses.

Although we did not observe the usual spatial distribution pattern typically associated with the oN1, we did find evidence supporting the presence of distinct specific and unspecific predictions in our paradigm. Specifically, the oN2 component exhibited a larger amplitude in the single sound condition compared to the random sound condition. This indicates that participants generated predictions about the specific identity of the upcoming stimulus when that was possible, and otherwise formed a more general prediction of producing any sound with their button press.

### 4.2 Readiness potential reflects identity-specific predictions

The RP has long been discussed to reflect preparatory processes of an upcoming motor task (Shibasaki & Hallett, 2006). Still, its neurophysiological implications have been the subject of a lively debate for decades. Based on the idea of a stochastic accumulator,Schurger et al. (2012)suggested that spontaneous fluctuations in neural activity influence the precise moment in time when a person moves voluntarily. When time-locked to movement onset, and averaged across repeated movements, these spontaneous fluctuations add up to a negative potential shift. This would implicate that the RP reflects accumulated noise, rather than specific, motor preparatory processes. However, recent work on the RP challenges this idea by demonstrating that the RP is larger in conditions in which movements have a predictable auditory or visual consequence, compared to no visual or auditory consequence (Reznik et al., 2018;Vercillo et al., 2018;Wen et al., 2018). This has been taken as evidence that the RP might not merely reflect preparatory motor activity, but also prediction of the upcoming sensory feedback expected for an imminent voluntary motor action, i.e., systematic mental processes, rather than spontaneous fluctuations.

The findings of our study support and extend the idea that the RP is influenced by predictive processing. Specifically, we observed a larger (more negative) amplitude in the single sound condition, compared to the random sound condition and motor control condition. Previous studies, such as those byReznik et al. (2018)andVercillo et al. (2018), have demonstrated that the RP is larger when visual or auditory feedback is provided. Additionally,Wen et al. (2018)found that the RP is larger when there is consistent auditory feedback, compared to inconsistent feedback, showing that the RP reflects the reliability of action consequences.Pinheiro et al. (2020)demonstrated that imagined and real auditory feedback had comparable effects on enhancing the RP’s amplitude compared to a motor-only condition. This may indicate that the observed RP effect relates to an “image”, in the ideomotor sense, of action effects, even if the subject knows that the effect will not really be (acoustically) present. The modulation of RP amplitude by (expected or imagined) sensory consequences may thus provide insight into phenomenal aspects of action-effect anticipation. This is also supported by the additional effect of complexity described byPinheiro et al. (2020). They report a further increase in RP amplitude when the expected, or imagined, sound is one’s own vocal, and therefore more meaningful and phenomenologically rich, compared to a meaningless sine-wave tone.

However, in these studies, the specificity with which sensory feedback could be predicted did not vary. The present study demonstrates that not the mere presence of auditory feedback, but the specific predictable identity of the auditory feedback, affects the RP. This indicates that the RP is not sensitive to predictions of any feedback, but is influenced by expectations about exactly what that feedback will be.

Bayesian statistics supported the null hypothesis of no difference in RP amplitude between the random sound and the motor control (no sound) condition. While subjects generated predictions even when sound identity could not be known a priori, evident from oN1 and oN2 results observed in the random sound condition, these general predictions do not seem to influence the RP. This can be considered in line with the idea of ideomotor theory, where movement initiation and selection are influenced by specific images of concrete action-effects (Shin et al., 2010).

On the other hand, effects of general prediction on the RP may be task dependent. If the task is to produce any sound (e.g., honk in traffic), general predictions may play an important role in movement preparation, and influence RP after all. The influence of specific prediction on the RP, on the other hand, seems to be mandatory, and independent of the task at hand. This is because effects of sound specificity in our study existed even though sound had no (explicit) task-relevance. It could be argued that people used sound in our study as auditory feedback to ensure that they maintained the correct pace of movement. This is possible, given that auditory input, in particular, carries valuable information about time (Bolognini et al., 2012). However, if RP is modulated because subjects use sound to pace their movements, one would have to assume that they do so only when sound identity can be predicted.

Our results align with the ideomotor theory and the forward model framework. According to the ideomotor theory, specific actions are generated through associations between stimuli, motor responses, and internalized images of anticipated action effects. These associations are reinforced through repetition and conditioning, ultimately giving rise to anticipatory images that govern the sequence of actions and action effects (Korka et al., 2022;Shin et al., 2010). The forward model framework (Friston, 2005;Schröger et al., 2015;Wolpert & Flanagan, 2001), on the other hand, suggests that there is a functional connection between actions and sensory outcomes via a predictive efference copy (von Holst, 1954) and corollary discharge (Crapse & Sommer, 2008). Both ideas, the idea of an anticipatory image and the idea of a motor-based prediction, are in line with our results.

The early RP, originating in higher-order motor regions such as the pre-SMA and SMA, has been linked to predictive processes (Pinheiro et al., 2020;Reznik et al., 2018;Wen et al., 2018). In the present study, cluster-based permutation testing revealed significant enhancements of both phases of the RP in the single sound condition compared to the random sound condition. The observed increase in early RP adds to the growing body of evidence, suggesting that the early RP and its associated brain regions play a crucial role in encoding the expected auditory consequences of self-generated actions (Reznik et al., 2018;Wen et al., 2018), even when sensory feedback is merely imagined (Pinheiro et al., 2020).

The late RP, more prominent over motor cortex, is usually associated with late-stage preparation of imminent movement (Shibasaki & Hallett, 2006). However, research exploring the influence of the primary and supplementary motor areas on sensory areas has revealed notable modulations in the auditory and visual cortices’ response to self-generated actions, such as sounds and visually cued movements, compared to passive perception of the same stimuli (Eisenberg et al., 2011;Reznik et al., 2015). These studies suggest that motor areas, particularly the primary motor cortex (M1) and supplementary motor areas (SMA), might play an important role in shaping sensory experiences, likely through predictive mechanisms that prepare the sensory system for expected consequences of movements. Our observation of an enhanced late RP is consistent with previous research exploring the role of RP in predictive processes (Pinheiro et al., 2020;Reznik et al., 2018). This suggests that predictive mechanisms may be encoded not only in the early RP but also in the late RP. However, further research is required to clarify the distinct contributions of the functional neuroanatomy of both lower and higher-level motor regions in encoding the sensory outcomes of voluntary actions.

Many factors, such as physical (e.g., speed, force, amplitude) and psychological (e.g. attention) aspects of a voluntary movement, have been shown to influence RP amplitude (Shibasaki & Hallett, 2006). It has also been shown that the amplitude of the RP varies depending on the time intervals between consecutive movements (Trovò et al., 2021;Verleger et al., 2016). However, in our study, inter-press intervals did not differ between conditions. Therefore, condition differences in RP amplitude in our study are not explained by differences in the timing of movements.

### 4.3 PMBR is not modulated by auditory feedback

PMBR amplitude has been associated with sensory prediction and its role in outcome evaluation and motor learning (Tan et al., 2014,Tan, Wade, et al., 2016,Tan, Fischer, et al., 2016). In general, we observed no significant differences in PMBR between auditory feedback conditions matched for physical events (i.e., between conditions with sound, and between conditions without sound) as indicated by repeated-measures ANOVA. The “omission”-ANOVA indicated a trend-level difference between conditions. Post-hoc testing pointed to a stronger PMBR for single omission trials, compared to random omission trials (*p*= 0.049); however, there was no significant difference between the random omission trials and the motor control condition. If the difference between random omission trials and single omission trials truly reflected different levels of reliability of sensory prediction, we would expect an even stronger difference between the random omission trials and the motor control condition. This is because the motor control condition allows for the most reliable sensory prediction across the three conditions (i.e., fully predictable absence of auditory feedback). However, PMBR in the random omission trials, that is, the least predictable trials in the experiment, was similar to the motor control condition, that is, the most predictable trials (Fig. 5A). This, together with the absence of any cluster in cluster-based permutation testing, casts doubt on the marginally significant difference in PMBR between random and single omission trials.

Our paradigm differed from the visuomotor learning studies that have demonstrated an effect of predictability on PMBR (Korka et al., 2023;Palmer et al., 2019;Tan, Wade, et al., 2016), in two fundamental aspects. First, the consequence of movement in our study was auditory, not visual. Therefore, it might be possible that a modulation of the PMBR is specific to visual (or somatosensory) feedback, or to sensory feedback that carries rich spatial information about the movement, more than binaurally presented sounds. Second, in previous studies, the presented visual stimuli were relevant to the execution of the motor task (Palmer et al., 2019;Tan et al., 2016), whereas in our study, sounds were largely irrelevant for the motor task. Therefore, our results suggest that the modulation of PMBR by altered sensory feedback may be bound to feedback that is task-relevant, spatial, and/or visual/visuo-proprioceptive (Korka et al., 2023). Studies in which auditory feedback is relevant for future movements are needed to further test these ideas.

## 5 Conclusion

Our study demonstrates that identity-specific predictions influence the preparation for movement as reflected in the RP amplitude, even when the predicted stimulus is not explicitly task-relevant. Our findings align with the ideomotor theory, which suggests that actions are generated based on associations between stimuli, motor responses, and anticipated action effects.

## Data Availability

Data and code access is granted upon request. However, a formal data-sharing agreement is required. Requests for data can be submitted viachristoph.a.gaertner@googlemail.com.
